# The Prevalence of Pain in Chronic Diseases: An Umbrella Review of Systematic Reviews

**DOI:** 10.3390/jcm12237302

**Published:** 2023-11-25

**Authors:** Dmitriy Viderman, Karina Tapinova, Mina Aubakirova, Yerkin G. Abdildin

**Affiliations:** 1 Department of Surgery, Section of Anesthesiology, Intensive Care, and Pain Medicine, School of Medicine, Nazarbayev University, Astana 010000, Kazakhstan; 2 School of Engineering and Digital Sciences, Nazarbayev University, Astana 010000, Kazakhstan

**Keywords:** chronic pain, prevalence, chronic diseases, cancer-related pain, outcomes, neurodegenerative diseases

## Abstract

Since pain is common in many diseases, it is important to summarize the precise prevalence data on pain and high-impact pain, which frequently worsens the quality of life and work activities. This umbrella review aims to estimate the prevalence of pain among patients with different chronic diseases/conditions. We followed the PRISMA guidelines. We identified the following areas addressing the prevalence of pain: (1) pain in cancer patients; (2) neurodegenerative diseases; (3) chronic heart failure; (4) chronic obstructive pulmonary disease; (5) chronic kidney diseases; (6) liver diseases and failure; (7) nursing home seniors; and (8) postamputation (phantom) pain. We included systematic reviews and meta-analyses that reported pain in patients from the mentioned populations. The prevalence of pain in chronic diseases is high, in some cases even higher than the cardinal symptoms of these diseases/conditions. Most patients who suffer from any of these diseases/conditions can develop chronic pain at later stages. Pain in chronic diseases does not receive enough attention and is not properly managed. Future studies are warranted to establish a more precise prevalence of chronic pain and develop better methods of pain screening, detection, and management.

## 1. Introduction

Pain is a significant public health issue with massive socio-economic impacts. Pain is one of the most common reasons why patients seek medical care [1]. It has been associated with limitations in mobility and everyday tasks, reliance on opioids, anxiety, and depression, as well as a lower perception of overall health and reduced quality of life [1,2,3,4]. 

Chronic pain is defined by the “International Association for the Study of Pain” (IASP) as “pain that persists or recurs for more than 3 months” [5]. The prevalence of pain among the adult population varies significantly, depending on the geographical area, age, and comorbidities [6]. Since pain is widespread in many diseases, it is important to summarize the precise prevalence data on pain and high-impact chronic pain, which frequently worsens the quality of life and work activities. The estimates of high-impact pain can help identify individuals whose major life domains, such as work, social life, recreation, and self-care activities, are affected by pain, providing valuable insights into the specific population in need of pain services [6].

Pain is a significant concern and is frequently reported as one of the most prevalent symptoms among patients presenting with oncological, neurological, musculoskeletal, cardiovascular, liver, pulmonary, and renal diseases and conditions. Inadequate relief can have demoralizing consequences, negatively affecting physical condition, overall quality of life, and emotional well-being. It often leads to anxiety, anger, depression, and even cognitive impairment [7,8,9,10].

There are numerous factors contributing to the lack of improvement in cancer pain management. Among the common barriers faced by healthcare professionals are lack of knowledge and skills in pain medicine, inadequate pain assessment practices, and reluctance to prescribe analgesics [11].

Conversely, patients have their own barriers, such as hesitance to discuss pain with physicians and nurses, reluctance to look for treatment, difficulties adhering to analgesic prescriptions, and depression as well as other cognitive and psychological factors [12,13]. 

Since numerous systematic reviews have been published and the studies on the prevalence of pain in chronic diseases are heterogeneous and broad, there is a need to synthesize existing evidence from multiple systematic reviews. To comprehensively assess the prevalence of pain in patients with chronic diseases, we undertook an “umbrella” review, which involved surveying existing systematic reviews and meta-analyses, representing one of the most robust forms of evidence synthesis. Our approach began with a scoping review to pinpoint areas of research consistently supporting the prevalence of pain among patients with different chronic diseases/conditions. We excluded all primary painful diseases, such as trigeminal neuralgia, low back pain, and osteoarthrosis. We identified the following areas addressing the prevalence of pain: (1) pain in cancer patients; (2) neurodegenerative diseases; (3) chronic heart failure; (4) chronic obstructive pulmonary disease (COPD); (5) chronic kidney diseases; (6) liver diseases and liver failure; (7) nursing home seniors; and (8) postamputation (phantom) pain. Therefore, the objective of this umbrella review was to estimate the prevalence of pain among patients from the mentioned populations. 

## 2. Materials and Methods

### Search Strategy and Selection Criteria

The current umbrella review was conducted following the PRISMA guidelines [14]. The protocol registration DOI is https://doi.org/10.17605/OSF.IO/RXVWA. We searched for systematic reviews, meta-analyses, and large database studies in eight areas using PubMed, Scopus, and the Cochrane database. The search encompassed studies published until March 2023. We searched for the prevalence of pain among the diseases and conditions associated with frequent pain including the prevalence of pain in (1) cancer patients, (2) neurodegenerative diseases, (3) chronic heart failure, (4) chronic obstructive pulmonary disease, (5) chronic kidney diseases, (6) liver diseases and failure, (7) nursing home seniors, and (8) postamputation (phantom) patients. The search terms and their combinations are described in the Supplementary File.

Additionally, specific terminology tailored to each field of research was employed. The inclusion criteria were devised to identify the most reliable evidence for each research domain and comprised the following:

1. Systematic reviews and meta-analyses focused on the prevalence of pain;
2. Studies published in peer-reviewed journals;
3. When more than five systematic reviews were available, we included the five most recent ones.

The exclusion criteria involved the following: (1) animal studies; (2) individual observational studies (not systematic reviews); (3) case reports; (4) editorials. No restrictions based on language or publication date were applied. 

Two authors conducted the search and screening independently. In case of disagreements, the rest of the authors were engaged in resolving the dispute. In instances where data were missing or unclear, we contacted the authors of the papers for clarification. We extracted summary effects, confidence intervals, and measures of statistical significance when available. Due to overlapping studies and variations in reporting styles and units, we did not conduct a meta-analysis for each area. All the extracted data are presented in Table 1 and Table 2. The quality of systematic reviews and meta-analyses was assessed using AMSTAR-2 (A MeaSurement Tool to Assess systematic Reviews) [15]. This tool consists of 16 questions related to the design and reporting of the study, to which one of the three possible answers is given: “Yes”, “No”, or “Partial yes”. This assessment does not imply an overall score but rather assesses each study on every one of the 16 domains.

## 3. Results

**Figure 1:**
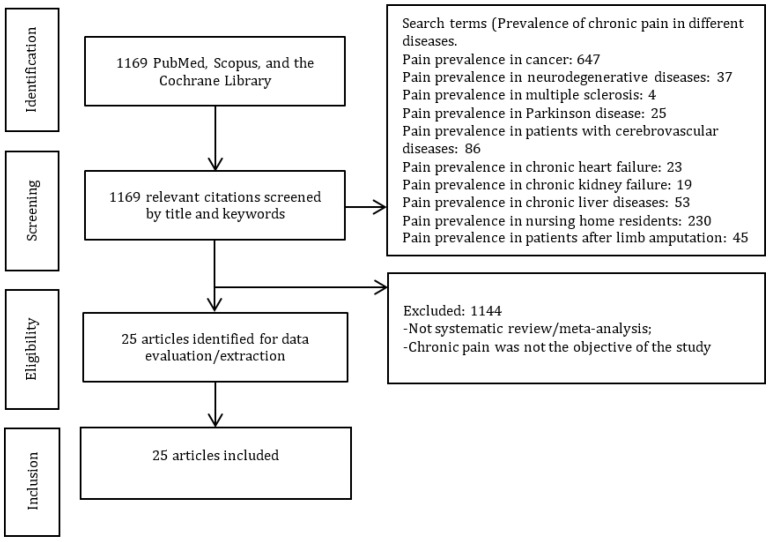
PRISMA diagram.

Twenty-five studies were included in this umbrella review (Figure 1).

### 3.1. Patient Characteristics

#### 3.1.1. Diseases/Conditions

Diagnosis in which the prevalence of pain was studied: We included systematic reviews and meta-analyses (SRs & MAs), which reported pain in patients with a wide variety of diseases and conditions including cancer (head and neck, bronchus/lung, breast, gastrointestinal tract, prostate, gynecological cancers, rectal), arthritis, neurodegenerative diseases, depression, dementia, osteoporosis, pressure ulcer, falls, anxiety, chronic heart failure, chronic obstructive pulmonary diseases, chronic kidney diseases and end-stage kidney failure, end-stage liver disease, traumatic brain injury, and cerebrovascular diseases (Table 1 and Table 2) [16,17,18,19,20,21,22,23,24,25,26,27,28,29,30,31,32,33,34,35,36,37,38,39,40].

#### 3.1.2. Countries (Where the Included Studies Were Conducted/Data Collected) 

The included systematic reviews and meta-analyses reported the prevalence of pain in patients from many geographical regions and countries (Table 1). Some individual systematic reviews studied the prevalence of pain in patients from several geographical regions including Europe, Asia, North America, South America, and Oceania representing the following countries: Finland, the Netherlands, Italy, Norway, the United States of America, Hong Kong, Czech Republic, England, France, Germany, Israel, Japan, Brazil, Turkey, Austria, Sweden, Australia, China, Korea, Denmark, the UK, Scotland, Greece, India, Egypt, Canada, Taiwan, Cyprus, Spain, Switzerland, Malaysia, Thailand, Nigeria, Saudi Arabia, Republic of Guinea, Morocco, South Korea, Uruguay, Lebanon, Poland, Iran, Sri Lanka, and Pakistan (Table 1).

### 3.2. Quality Assessment

AMSTAR evaluation was used for quality assessment (Table 3). All the studies included the components of PICO in their research questions and inclusion criteria. Seven studies explicitly stated that a protocol was developed prior to conducting the study and that corresponding changes were made to the protocol. Five studies explained the study design selection. Seventeen studies mentioned conducting the study selection in duplicate, and only four studies mentioned extracting the data in duplicate. Only two studies provided a list of excluded studies with justifications. Most studies had some descriptions of the included studies. If a meta-analysis was performed, all the studies used appropriate statistical methods. However, none of the studies discussed the impact of the risk of bias of individual studies on the results, and few studies discussed heterogeneity. Six studies examined publication bias. A few studies did not mention conflict of interest, and none of the studies considered funding of the included studies.

### 3.3. Prevalence of Chronic Pain in Different Diseases/Conditions

In total, out of the 25 included studies, we included 4 that reported pain in cancer patients [16,17,26,31]; 7 that reported pain in neurodegenerative diseases including amyotrophic lateral sclerosis (ALS), multiple sclerosis, Parkinson’s disease, Huntington’s disease, and dementia [20,21,22,23,24,25,40]; 2 that reported pain in cerebrovascular diseases [38,39]; 1 that reported pain in chronic heart failure (CHF) [28]; 4 that reported pain in chronic kidney disease (CKD) [32,33,34,35], 1 that reported pain in traumatic brain injury (TBI) [37]; 2 that reported pain in COPD [29,30]; 1 that reported pain in chronic liver diseases [36]; 2 that reported pain in nursing home residents and seniors [18,27]; and 1 that reported pain in chronic pain after limb amputation [19].

#### 3.3.1. Prevalence of Chronic Pain in Cancer Patients

**Figure 2:**
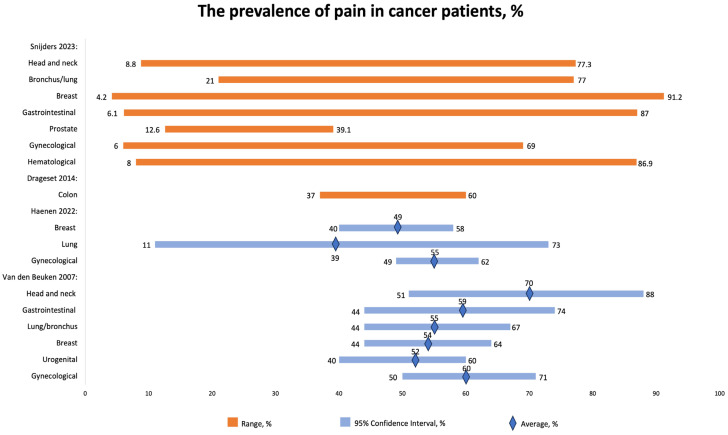
The prevalence of pain in cancer patients, %. CI, confidence interval. Sniders 2023 [16], Drageset 2014 [26], Haenen 2022 [17]; Van den Beuken 2007 [31].

We included four studies that reported the prevalence of chronic pain in cancer patients (Figure 2). The reported prevalence varied relatively widely, mostly depending on the stage of cancer, rather than the type of cancer.

Snijders 2023 [16] studied the prevalence of chronic pain in cancer patients. The overall prevalence of cancer-related pain was reported to be 44.5%. Importantly, among treatment groups, the lowest prevalence (35.8%) was observed in groups that received curative treatment. Overall, the prevalence of pain in cancer patients remains high, especially in metastatic, advanced, and terminal patients, reaching 54.6%. The prevalence of pain was as follows: the highest prevalence was reported in patients with breast cancer (4.2–91.2%), followed by hematological malignancies (8–86.9%), GI (6.1–87%), head and neck (8.8–77.3%), bronchus/lung (21–77%), gynecological (6–69%), and prostate (12.6–39.1%).

The authors reported that a reduction in the prevalence and intensity of cancer-related pain might be positively influenced by the improvement in awareness and knowledge among healthcare professionals, treatment strategies, and new pain management guidelines. Nevertheless, disparities in knowledge among healthcare professionals from different countries should be addressed. The prevalence of cancer-related pain was significantly higher in Asia, South America, and Africa compared to Europe. Many cancer patients in low-income countries receive inadequate pain control, partially due to inadequate education. Moreover, doctors generally possess more advanced knowledge about pain management compared to nurses; however, both have an insufficient understanding of pharmacology, especially the side effects of opioids. Furthermore, the majority of oncology nurses have insufficient knowledge in this area [16].

Haenen et al. [17] reported the following prevalence of pain according to the type of cancer: the highest prevalence was registered in gynecological cancer (55% (95% CI 49–62%)), followed by breast cancer (49% (95% CI 40–58%)) and lung cancer (39% (95% CI 11–73%)). At least half of cancer patients experience pain at least three months after therapy.

About half of breast cancer survivors experience pain, with estimates ranging from 40% to 58%. Similarly, around 39% of lung cancer survivors and 55% of gynecological cancer survivors also report experiencing pain, with confidence intervals of 11% to 73% and 49% to 62%, respectively.

Around 47% of individuals reported pain more than three months after successful completion of cancer treatment. However, there is insufficient evidence to determine whether the type of cancer, treatment approach, method of pain measurement, or duration of follow-up have any significant impact on the occurrence of pain in these cancer survivors [17].

Van den Beuken-van Everdingen et al. (2007) [31] also reported the prevalence of pain in patients with different types of cancers, including head and neck cancer (70% (51–88)), GI tract (59% (44–74)), lung/bronchus (55% (44–67)), breast (54% (44–64)), urogenital (52% (40–60)), and gynecological (60% (50–71%)).

Cancer pain can be generated by (1) cancer itself or metastases (infiltration of soft tissues, bony metastasis, and compression of nerves); (2) treatment-related side effects (chemotherapy-induced musculoskeletal pain, mucositis); (3) postoperative pain; and (4) radiation-induced dermatitis, mucositis, or enteritis. Pain may become chronic in many cancer survivors. Some chemotherapeutic agents, such as platins, bortezomib, taxanes, or vincristine, can cause painful chemotherapy-induced peripheral neuropathy, whereas aromatase inhibitors are associated with diffuse joint pain. As described earlier, surgery can result in phantom limb pain or pain from scar formation [41,42,43,44].

#### 3.3.2. Prevalence of Pain in Nursing Home Residents

**Figure 3:**
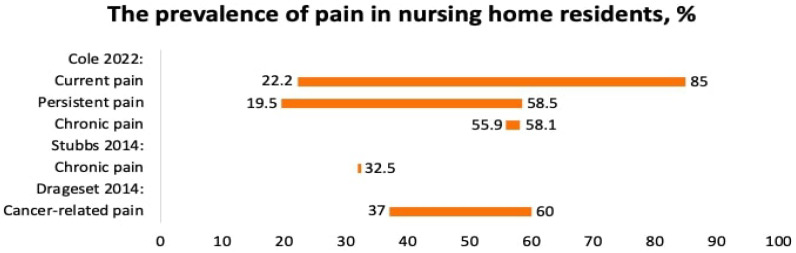
The prevalence of pain in nursing home residents, %. Cole 22 [18], Stubbs [27], Drageset 2014 [26].

Three systematic reviews reported the prevalence of chronic pain in seniors and nursing home residents [18,26,27]. Cole et al. (2022) reported the prevalence of pain among nursing home residents with different comorbidities including arthritis, depression, dementia, osteoporosis, pressure ulcers, falls, anxiety, and contractures [18]. Since the residents suffered from several diseases, it was not possible to find out precisely what disease and condition caused the pain. The prevalence of “current pain” at the time of the survey varied from 22.2 to 85%, whereas persistent pain varied from 19.5 to 58.5%, and chronic pain varied from 55.9 to 58.1% (Figure 3). 

Drageset et al. (2014) reported the prevalence of cancer-related pain in nursing home residents [26]. The prevalence of pain varied from 37 to 60%.

Stubbs et al., 2014, reported the prevalence of chronic pain among seniors from the US, the UK, Australia, Italy, Japan, China, Nigeria, the Netherlands, Taiwan, and Turkey. The prevalence of pain was 32.5% (Figure 3). Seniors with pain were more likely to fall, especially with foot pain [27].

Many residents suffered from depression, which could worsen the pain severity or be triggered by pain. Moreover, depression, limitations in daily activities (immobility), cognitive impairment, dementia, and arthritis were consistently associated with pain. Dementia and cognitive dysfunction impair the ability to communicate and report pain. Subsequently, pain in non-verbal residents is usually detected from facial expressions, crying, and guarding of body parts. Nursing home residents who cannot report the pain verbally may be experiencing pain that is not always recognized [18]. 

#### 3.3.3. Residual Limb Pain after Amputation

We included one study, that of Evans et al., 2022 [19], which reported the prevalence the residual limb pain after amputation due to cancer, trauma, and vascular pathologies.

Individuals who underwent limb amputation frequently experience postamputation pain. Among them, patients who had amputations due to cancer tend to report the highest pain intensity, followed by patients with traumatic amputations and amputations due to peripheral vascular diseases. Pain intensity in upper extremity amputations was reported to be higher compared to that in lower extremity amputations. Most individual studies included in the SR indicated that nearly all patients who underwent amputation had pre-amputation pain.

**Figure 4:**
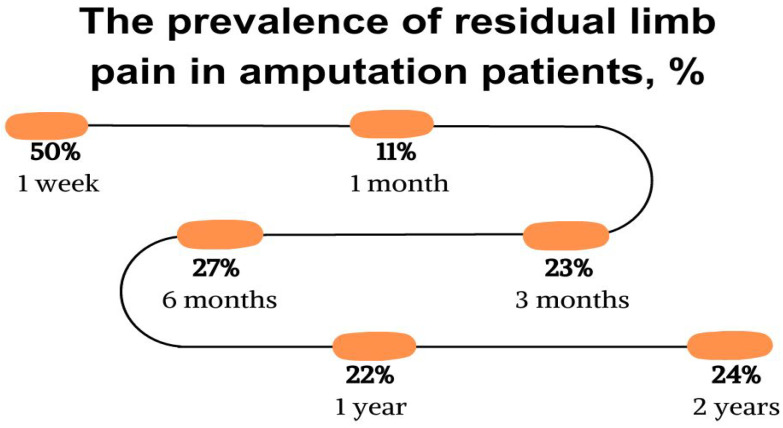
The prevalence of residual limb pain in amputation patients, %. Evans 2022 [19].

The pain occurs despite attempts to control it with different analgesic methods. From three months to two years, the prevalence of residual limb pain ranges between 22% and 27% (Figure 4). Individuals with amputations due to cancer often report the highest pain severity. It should also be noted that the reported pain severity is generally higher in individuals undergoing upper extremity amputations compared to lower extremity amputations, but this may be influenced by an increased presence of cancer or trauma in the studies involving upper extremity amputations [19].

When it comes to perioperative pain control, there is no consensus on the optimal approach. However, several aspects, such as the duration, route of administration, and content of perioperative prescribing practices, have been previously studied. For example, patients receiving epidural bupivacaine with morphine for three days preoperatively reported no residual limb pain at six and twelve months following amputation. Conversely, a three-day duration of early postoperative perineural and subfascial ropivacaine did not decrease residual limb pain severity at one month or longer follow-up. Comparisons have also been made between different pain control methods. It was also reported that epidural blocks led to decreased residual limb pain at one week compared to perineural blocks. It was also reported that among various methods of preoperative pain control, the group treated with epidural pain control throughout the perioperative period had a significant reduction in phantom limb pain severity at six months [19].

Additionally, studies have examined the use of adjunctive medications, such as calcitonin and clonidine, in combination with nerve blocks. Some of these medications showed improvements in reducing residual limb pain, while others did not yield significant benefits. Several medications, including gabapentin and valproic acid, have been tested for their effectiveness in reducing residual limb pain, but the results have been inconclusive. These studies primarily enrolled patients with amputations due to vascular diseases, so further investigation is needed to determine the efficacy of these medications in other subgroups or causes of amputation [19].

#### 3.3.4. Prevalence of Pain in Patients with Neurodegenerative Diseases

We included seven systematic reviews that focused on neurodegenerative diseases including amyotrophic lateral sclerosis (ALS), multiple sclerosis, Parkinson’s disease, Huntington’s disease, Alzheimer’s syndrome, and dementia (non-specified) [20,21,22,23,24,25,40].

**Figure 5:**
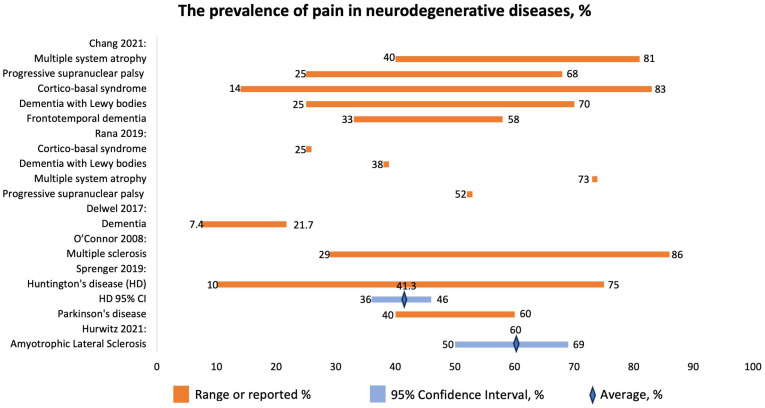
The prevalence of pain in neurodegenerative diseases, %. Chang 2021 [22], Rana 2019 [24], Delwel 2017 [25], O’Connor 2008 [40], Sprenger 2019 [23], Hurwitz 2021 [20].

The study by Hurwitz 2021 [20] that examined pain among ALS patients included several countries (Japan, US, Italy, Germany, Canada, France, Australia, Sweden, Finland, UK). The overall prevalence of pain (Figure 5) was 60% (95% CI 50–69%). The prevalence of pain in the head, neck, trunk, and back was almost 25%, while that for upper limbs was over 40%. The authors conclude that pain in ALS is high.

Chang 2021 reported the prevalence of pain in patients with atypical parkinsonism, MSA, PSP, CBS, DLB, and FTD (Figure 5) [22]. The disorders have been associated with the following pain locations and types:

- MSA: neck, back, limbs; MSK, neuropathic, dystonic;
- PSP: limbs, neck, back, MSK;
- CBS: limbs, dystonic;
- DLB: multiple places, neuropathic, MSK;
- FTD: head, neck, shoulder, abdomen, MSK.

Neurodegenerative diseases can lead to chronic pain. However, its prevalence might be underestimated due to the cognitive decline of patients. Patients with neurodegenerative diseases, such as atypical parkinsonism, may have a higher likelihood of pain. Since the pathophysiology of pain in these diseases might include different pathways and mechanisms, it is important to use a strategy based on understanding the underlying mechanisms when choosing the most suitable treatment for pain relief. However, there is insufficient evidence regarding the exact physiological mechanisms that contribute to pain in specific neurodegenerative disorders. Furthermore, some patients may have difficulty accurately reporting pain due to cognitive issues or changes in speech and language, which can result in unreliable findings when using clinical assessment tools or quantitative sensory testing in research studies [22]. 

Multiple system atrophy (MSA) is defined as a neurodegenerative disease characterized by the presence of α-synuclein-positive oligodendroglial cytoplasmic inclusions and degenerative changes in certain brain structures. It can manifest with predominant parkinsonism (MSA-P) or predominant cerebellar features (MSA-C), along with various autonomic and pyramidal symptoms. Pain is a common non-motor symptom in MSA, with chronic musculoskeletal pain being the most frequently reported subtype. Neuropathic pain, both central and peripheral, is also prevalent in MSA. The severity of pain tends to increase as the disease progresses. The exact underlying causes of pain in MSA are not fully understood, but there are pathological changes in brain regions associated with pain processing. Some MSA patients may also experience peripheral neuropathy, which contributes to neuropathic sensations. Pain treatment in MSA patients is often inadequate, with a significant number not receiving proper pain-relieving therapies. Dopaminergic therapy may provide partial relief for some patients, but it does not consistently improve pain sensitivity. Botulinum toxin injections have shown some effectiveness in relieving pain associated with dystonia and sialorrhea. Physiotherapy and medications like pregabalin, gabapentin, and amitriptyline may be beneficial for neuropathic pain, although more high-quality evidence is needed. Progressive supranuclear palsy (PSP), another neurodegenerative syndrome, also presents with pain, but the prevalence appears to be lower compared to MSA and Parkinson’s disease (PD) according to questionnaire-based studies [22].

Sprenger et al. (2019) [23] studied the prevalence of pain in patients with HD. The prevalence of pain was 41.3% (95% CI: 36–46%) with a range of 10–75% (Figure 5). Pain might be one of the HD symptoms. 

Huntington’s disease (HD) is a devastating neurodegenerative condition, inherited in an autosomal-dominant manner. It leads to a range of distressing symptoms, including characteristic motor symptoms, such as chorea, as well as cognitive, emotional, and behavioral disturbances. Secondary symptoms may include weight loss, sleep disorders, and autonomic problems. HD causes significant atrophy in the brain, especially affecting the GABAergic system. Interestingly, this striatal atrophy is already evident during the pre-manifest stage, which can be up to 10 to 15 years before the actual clinical diagnosis. As the disease progresses to the manifest stage, the severity of atrophy in the striatum correlates with the overall disease severity; total functional capacity; and cognitive disturbances, including disturbances affecting memory, executive function, and processing speed. The striatum is part of the cerebral “pain matrix”, a network responsible for various aspects of pain processing, such as its sensory-discriminative, affective-emotional, and cognitive-evaluative dimensions. Notably, the striatum seems to be predominantly involved in the affective-motivational and cognitive-evaluative dimensions of pain, which play a crucial role in understanding pain; suffering level; unpleasantness; and the ability to remember, interpret, and respond appropriately to pain. Apart from its role in pain processing, the striatum also serves an analgesic function. Experiments involving morphine injections into the marginal divisions of the striatum resulted in dose-dependent hypoalgesia, which could be reversed by naloxone. Additionally, the striatum contains a high concentration of endogenous opiates and their receptors. Other brain regions in the “pain matrix” and their associated pain dimensions include the primary as well as secondary somatosensory cortices (sensory-discriminative), anterior cingulate cortex (affective and cognitive), thalamus (sensory-discriminative and affective), amygdala (affective), insula (affective and cognitive), and prefrontal cortex (affective and cognitive). Studies using magnetic resonance imaging have found atrophy in these regions in both the premanifest and manifest stages of HD, with disease progression correlating with increased atrophy. Research on pain in HD patients has yielded conflicting results. Some studies showed that a significant number of patients experienced pain, but not all of them received appropriate analgesic treatment [22].

The prevalence of pain in patients with Huntington’s disease (HD) was 41.3%, compared with 40–60% in another neurodegenerative disease, Parkinson’s disease. Additionally, the prevalence of pain could be influenced by various factors such as age; sex; drug treatment; motor functions; cognitive, emotional, and behavioral disturbances; comorbidities; the severity and duration of the disease; and the site and types of pain (e.g., nociceptive vs. neuropathic and acute vs. chronic pain). Moreover, higher scores of depression and anxiety, the use of analgesic medication, and the presence of comorbid conditions were linked to an increased likelihood of experiencing more severe pain in HD patients [23].

The symptoms and worries experienced by patients with HD differ as the disease progresses. In the early stages, known as premanifest HD, many patients express concerns related to their social interactions, such as complex family relationships and lack of support from their environment. On the other hand, during the manifest stage, physical issues become more prominent, including difficulties with swallowing food, driving, and walking. As a result of the significant impact of HD on various aspects of health, pain may play a relatively smaller role in affecting patients’ quality of life, and consequently, it is less commonly reported [23].

Rana et al., 2019, focused on the prevalence of chronic pain in patients with atypical parkinsonism [24]. They report prevalence for CBS to be 25%; for LBD, 38%; for MSA, 73%; and for PSP, 52% (Figure 5).

##### Characteristics of Pain

Patients with atypical parkinsonism experience pain, especially in limbs [21].

Delwel et al., 2017, studied the prevalence of orofacial pain in patients with dementia [25]. Older individuals suffering from dementia exhibit more significant oral health issues compared to those without dementia. These issues include problems such as coronal caries, root caries, and retained roots. However, it was observed that the number of remaining teeth and the decayed missing filled teeth index were similar between both groups. There is a lack of research on orofacial pain in older people with dementia. As a result, there is an urgent need for further investigation and focus on oral health, particularly concerning orofacial pain, in this vulnerable population.

O’Connor et al. (2008) focused on the prevalence of chronic pain in patients with multiple sclerosis (MS) [40]. The prevalence of pain ranged between 29 and 86%. The following types of pain were reported: extremity, back pain, headache, trigeminal neuralgia, neuropathic, MSK, and mixed.

The evidence available indicates that pain is prevalent in individuals with MS. It affects around one in five patients at the onset of the disease, about half of patients at any given time during their illness, and up to three-quarters of patients within the last month. This pain has a significant impact on the health-related quality of life, leading to impairments in both physical and emotional functioning. MS-related pain can be categorized into various types, including continuous central neuropathic pain, intermittent central neuropathic pain, musculoskeletal pain, and mixed neuropathic and non-neuropathic pain [40]. It is crucial to differentiate between these pain types in future studies. To achieve this, clear descriptions of the pain assessment methods and the timeframe for pain evaluation should be provided. Comparing the pain experiences of MS patients with those of non-MS patients having similar disability and healthcare utilization can help determine the extent to which pain is specifically attributable to MS. Depression and psychosocial risk factors have been linked to MS-related pain, but it remains uncertain whether they are causal factors for pain development or consequences of pain [40].

#### 3.3.5. Prevalence of Pain in Chronic Heart Failure Patients

**Figure 6:**
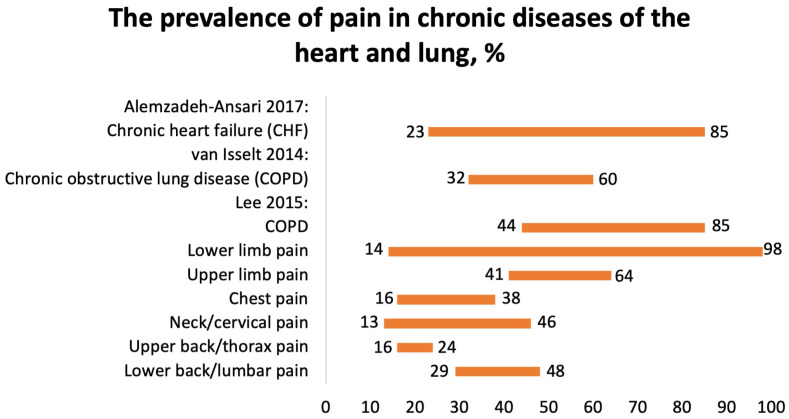
The prevalence of pain in chronic diseases of the heart and lung, %. Alemzadeh-Ansari 2017 [28], van Isselt 2014 [29], Lee 2015 [30].

One systematic review, conducted by Alemzadeh-Ansari (2017), reported the prevalence of pain in patients with chronic heart failure (CHF) [28]. The prevalence of pain in 20,875 patients with CHF varied from 23 to 85% (Figure 6). The most commonly reported types of pain were chest pain and neuropathic pain. The mechanisms of pain included ischemia, inflammation, ascites, and constipation [28]. 

The mechanisms of pain in CHF patients are not fully understood. Pain perception may vary and be altered by other symptoms such as fatigue, dyspnea, anxiety, and depression. Since approximately 80% of patients with CHF are elderly, several sources of pain, including psychological, physical, and neurological diseases, may contribute to the prevalence of pain. Some comorbidities, such as malignancies, coronary artery disease, COPD, pneumonia, peripheral vascular disease, depression, diabetes mellitus, osteoarthritis, and low back pain, are also common in CHF patients and can be the source of chronic pain [28]. 

#### 3.3.6. Prevalence of Pain in COPD

We included two SRs focusing on the prevalence of pain in patients with COPD. Van Isselt (2014) reported the prevalence of chronic pain in patients with COPD [29]. Pain is a significant issue in patients suffering from COPD, affecting an estimated 32–60% of individuals (Figure 6). However, little is known about the factors contributing to pain, and there is a dearth of information regarding interventions aimed at alleviating pain in COPD patients. Studies have indicated that pain is more prevalent in patients with moderate airflow limitation compared to those with severe or very severe airflow limitation. 

According to the SR by Lee et al. (2015), the polled prevalence of pain in 8677 COPD patients was 66% (95% CI 44–85%) (Figure 6) [30]. It was localized in the shoulder, neck, upper limb, chest, lower limb, head, buttocks, and back. Higher pain intensity was associated with dyspnea, altered respiration mechanics, fatigue, poorer quality of life, and a greater quantity of comorbidities. Common pain regions were the lower limb (14–98%), upper limb (41–64%), chest (16–38%), neck/cervical (13–46%), upper back/thorax (16–24%), and lower back/lumbar (29–48%). The majority of patients with COPD and pain had at least one comorbidity. Common comorbidities included cancer, musculoskeletal, endocrine disorders, and depression. There were no differences in pulmonary function in patients with COPD with or without pain. 

Several factors may influence pain experience. Thus, comorbidities may be one of the influencing factors. The prevalence of comorbidities is higher in patients with COPD experiencing pain than in COPD patients without pain or healthy control individuals. Breathlessness is also associated with higher pain intensity and may be related to the respiratory mechanics observed in patients with COPD. Altered respiration and muscle overload have been associated with pain including low back pain. The relationship between breathlessness and pain is complex, involving common neural pathways, and is responsible for the discomfort of both symptoms [28,29]. 

#### 3.3.7. Prevalence of Pain in Chronic Kidney Diseases

**Figure 7:**
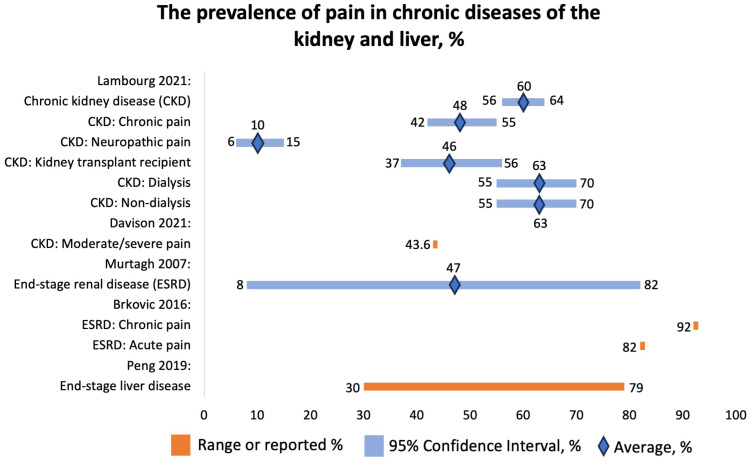
The prevalence of pain in chronic diseases of the kidney and liver, %. Lambourg 2021 [33], Davison 2021 [32], Murtagh 2007 [35], Brkovic 2016 [34], Peng 2019 [36].

We analyzed four SRs that studied the prevalence of pain in patients with chronic kidney disease (CKD). Davison et al. (2021) reported that 43.6% patients with CKD suffered from moderate or severe pain based on data from 16,558 patients (US, Saudi Arabia, Norway, Republic of Guinea, Italy, Morocco, Australia, Spain, South Korea, Hong Kong, Canada, Brazil, Turkey, Israel, Uruguay, Switzerland, Lebanon, Taiwan, Poland, UK, India, Netherlands, Iran, Sri Lanka, Malaysia, Germany, China) [32]. Of them, 60.5% were hemodialysis patients (Figure 7). The pain was localized in bones, joints, muscles, lower extremities, neck and shoulders, back, or upper extremities or was widespread. The reported mechanism of pain was neuropathic, “atheropathic”, or musculoskeletal. The research findings demonstrated that chronic pain was highly prevalent. Most patients experiencing pain rate its intensity as either moderate (typically ranging from 4 to 6 out of 10) or severe (ranging from 7 to 10 out of 10). Limited data exist for patients on peritoneal dialysis and those conservatively managed without dialysis, as well as for patients with CKD who did not require renal replacement therapy at the time of study. Patients managed conservatively reported the lowest prevalence of severe pain, likely due to “active” pain management in CKD. Interestingly, pain intensity rates were also high in patients with earlier stages of CKD and did not seem to correlate with the severity of their CKD, possibly indicating that the pain is mostly induced by comorbidities rather than CKD itself. Nonetheless, pain management is crucial for patients with CKD. Routine screening for pain in all CKD patients should be integrated into nephrology care. Pain assessment and management should be viewed as a component of quality care for CKD and chronic kidney failure patients [24,25,26,27,28,29,30,31].

Lambourg et al. (2021) concluded that 60% (95% CI 56–64%) of CKD patients experienced pain based on data from 40,678 individuals (Figure 7) [33]. Of them, 48% (95% CI 42–55%) reported chronic pain; 10% (95% CI 6–15%), neuropathic pain. Forty-six percent (95% CI 37–56%) of kidney transplant recipients and 63% (95% CI 55–70%) of dialysis patients reported pain. The most commonly reported types of pain included abdominal, headache, joint, and bone. The mechanisms of pain were neuropathic, postsurgical, and due to drug toxicity and mineral turnover.

Brkovic et al. (2016) found that among 6917 patients with end-stage kidney disease on hemodialysis, the prevalence of acute pain was 82%, and that of chronic pain was 92% (Figure 7) [34]. The cohort consisted of patients from Scotland, Italy, Kuwait, Brazil, the US, Canada, Morocco, Spain, the UK, Serbia, Turkey, Switzerland, Israel, Pakistan, Japan, Iran, and France. Patients most commonly reported headache, MSK, and diffuse pain. The most commonly mentioned mechanisms were ischemic and neuropathic. The pain was probably due to comorbidities and procedures.

Murtagh et al. (2007) reported that the prevalence of pain in ESRD was 47% (8–82%) (Figure 7). The pain was mainly caused by muscle cramps and headaches [35]. Apart from that, the authors also reported the prevalence of other symptoms, such as fatigue, 71% (12–97%); pruritus, 55% (10–77%); constipation, 53% (8–57%); anorexia, 49% (25–61%); sleep disturbance, 44% (20–83%); anxiety, 38% (12–52%); dyspnea, 35% (11–55%); nausea, 33% (15–48%); restless legs, 30% (8–52%); and depression, 27% (5–58%).

#### 3.3.8. Prevalence of Pain in Patients with Liver Diseases

We included one SR conducted by Peng et al. (2019) that focused on the prevalence of pain in end-stage liver disease [36]. The cohort consisted of 5434 patients from North America, Europe, Asia, and Africa. The reported prevalence of pain ranged from 30% to 79% (Figure 7). Interestingly, the prevalence of pain was greater than the prevalence of other symptoms, such as dyspnea (20–88%), muscular cramps (56–68%), nighttime insomnia (26–77%), daytime sleepiness (29.5–71%), anxiety (14–45%), depressive symptoms (4.5–64%), and erectile dysfunction (53–93%). The symptoms of end-stage liver disease are similar to the symptoms of other advanced diseases.

#### 3.3.9. Prevalence of Pain in Patients with Traumatic Brain Injury (TBI)

Only one SR that reported the prevalence of pain in patients who sustained TBI matched the criteria of our umbrella review. Nampiaparampil et al. (2008) conducted a comprehensive SR focusing on the prevalence of chronic pain including headaches, discussed other potential pain syndromes in patients with TBI, described the relationship between severity of brain injury and pain, and compared the civilian vs. combat veteran status on chronic pain [37].

**Figure 8:**
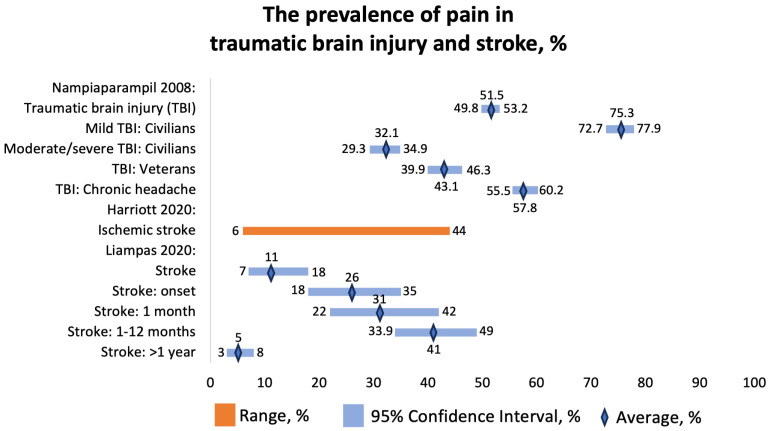
The prevalence of pain in traumatic brain injury and stroke, %. Nampiaparampil 2008 [37], Harriott 2020 [38], Liampas 2020 [39].

The prevalence of chronic pain was 51.5 (95% CI 49.8–53.2%) among civilians and 43.1 (95% CI 39.9–46.3%) among veterans (Figure 8). The prevalence of headache was 57.8 (95% CI 55.5–60.2%). Most headaches (51%) were localized in the occipital region. However, no significant association was found between the location of head trauma and the site of pain in these TBI patients. Apart from post-TBI headaches, these patients were found to develop complex regional pain syndrome and muscular-spasticity-related pain.

Interestingly, patients with mild TBI had a higher prevalence of chronic pain compared with those with moderate or severe TBI, and the reason for that remains unclear. It might be due to difficulty in reporting or processing symptoms, executive dysfunction, memory disturbances, and language deficits in patients with severe TBI. 

Chronic pain is a frequent consequence of TBI contributing to poor recovery. Patients who sustained TBIs in combat situations had higher rates of pain compared with the general population. Post-TBI patients might benefit from early screening, detection, and management of pain to reduce morbidity.

#### 3.3.10. Prevalence of Pain in Cerebrovascular Diseases

We included two SRs that focused on pain in patients with stroke. Harriott et al. (2020) found that the prevalence of stroke onset and poststroke headaches in 33,231 patients with ischemic stroke ranged from 6 to 44% (Figure 8) [38]. The mechanism of headaches included changes in innervation, increases in compartmental pressures, and meningeal inflammation. The importance of poststroke headaches is not given enough attention. There is a limited understanding of the predictive potential and treatment of new-onset poststroke headache or headache as a symptom of stroke. Some cerebrovascular conditions, such as venous sinus thrombosis or cervical artery dissection, have been well described as presenting with headaches, but few studies focus on new-onset and persistent headaches specifically after ischemic stroke. A new-onset headache during acute ischemic stroke was found to be a predictor of persistent headache six months after the stroke, and a poststroke headache is a common form of chronic poststroke pain, which can significantly impact disability. The overall prevalence suggests that around 14% of adult patients with ischemic stroke experience headaches either at the time of the stroke or shortly afterward. These headaches often persist for months to years, ranging from moderate to severe intensity, leading to significant disability associated with the condition. Several factors seem to be associated with poststroke headaches, including younger age, female sex, nonlacunar cortical stroke syndromes, and involvement of the posterior fossa. It appears that there may be differences in trigeminal and autonomic innervation of the posterior cerebral vessels, possibly explaining why posterior circulation strokes are more associated with headaches. The exact mechanisms underlying poststroke headaches are not clear, but they appear to be qualitatively different from typical migraines, even in individuals with a history of migraines. Possible factors contributing to poststroke headaches could be related to trigeminovascular afferents or meningeal inflammation, mechanical forces, spreading depolarization, platelet-derived factors, serotonergic mechanisms, or peptide-containing nociceptors, although more research is needed to fully understand these mechanisms [37].

Liampas et al. (2020) reported the prevalence of central poststroke pain (CPSP) at different time points following stroke [39]. The overall prevalence was 11 (95% CI 7–18%), at stroke onset, 26% (95% CI 18–35%); at 1 month following stroke, 31% (95% CI 22–42%); from 1 month to 1 year, 41% (95% CI 33.9–49.0%); and more than 1 year after stroke, 5% (95% CI 3–8%) (Figure 8). They included patients with strokes of various anatomical locations, including medullary stroke, thalamic stroke, somatosensory tract stroke, and cortical stroke. The pooled prevalence of CPSP in patients with medullary stroke was 57% (95% CI 24–85%, *n* = 216). The pooled prevalence of CPSP in patients with thalamic stroke was 52% (95% CI 41–62%, *n* = 93). The prevalence of CPSP was 53% (n = 30) in patients with stroke at any site of the somatosensory tract and 17% (*n* = 24) among patients with stroke with a cortical localization [38].

## 4. Discussion

### 4.1. Brief Summary

The objective of this umbrella review was to synthesize and analyze the highest level of available evidence on the prevalence of pain among patients with different diseases and conditions. It appears that pain does not receive enough attention, although it is highly prevalent in patients with heart, liver, and kidney failure; COPD; multiple sclerosis; Huntington’s disease; Parkinson’s disease; Alzheimer’s disease; traumatic brain injury; and cancer. Consequently, it is not properly managed, leading to patient suffering, which could be avoided by proper pain detection and treatment. In patients with certain diseases, pain is reported more frequently than the symptoms of the patient’s primary disease. Thus, the prevalence of cough, one of the leading symptoms in patients with COPD, was reported in about 55.5–65.6% of patients, and the polled prevalence of pain in COPD patients was 66% (95% CI 44–85%) [30]. In the North American patient population, the prevalence of dyspnea at rest, one of the most common symptoms of CHF, was 38% [45], whereas the prevalence of pain in CHF patients varied from 23 to 85%. Again, the prevalence of pain was probably even higher than the prevalence of the leading symptom of CHF. The same tendency is observed in other diseases.

### 4.2. Limitations

Many SRs included in this umbrella review did not provide detailed information about the intensity of pain or only presented an overall percentage of pain without specifying the different types of pain experienced. Some studies did not mention the specific type of pain, whether it was nociceptive, neuropathic, or a combination of both. The lack of consistent reporting of pain intensity limits the ability to assess the dimensions of pain. Many studies used different terms for specification of the duration of pain, such as simply “pain”, “chronic pain”, “residual pain”, and “recurrent pain”, and did not mention the duration of pain (e.g., 3 months). Greater evidence of the duration and frequency of pain episodes is needed to fully appreciate the pain experience. Several pain management options have been suggested; however, their effectiveness was not assessed. Therefore, further research is required to evaluate the effectiveness of possible interventions for pain management (COPD) [28]. The following subsections present limitations related to specific patient populations and recommendations for future studies.

#### 4.2.1. Nursing Home Residents

The prevalence and types of pain differ from study to study, possibly due to differences in research methodologies and objectives, the source of data, and the intensity of pain measured. Some studies did not report the duration of pain evaluation (days, months); therefore, it was challenging to classify the pain according to the chronology and analyze the impact of pain. Many studies did not provide the precise prevalence of pain in certain patient populations, such as patients with dementia or elderly nursing home residents, because they could not properly report the pain due to their cognitive abilities. 

To improve the ability to predict, detect, and manage pain among some vulnerable patient populations, such as nursing home residents and patients with dementia, it is crucial to study factors that increase the likelihood of pain reporting. Although dementia may affect pain thresholds and experience, resulting in a lower prevalence of pain, the most likely reason for the underestimated prevalence of pain is reduced reporting or detection of pain. Assessing pain in movement, rather than at rest, can probably better detect pain experiences, especially if relying on proxy reports. Using protocols that involve pain assessment during body movements may result in a more adequate determination of the prevalence of pain [18].

#### 4.2.2. Postamputation Pain

The rate of postamputation pain is one of the highest among all types of chronic postoperative pain. Overall, the optimal protocol for pain management during the perioperative period remains uncertain and requires more research. To reduce the prevalence of postamputation pain, future studies should focus on exploring preoperative pain control, surgical techniques, and postoperative pain management. Additional clinical trials with a low risk of bias are necessary to study the impact of multiday preoperative and postoperative nerve blocks on neuroma [19].

#### 4.2.3. Neurodegenerative Diseases

The prevalence of chronic pain in MS patients, especially at later stages, is high, affecting the majority of patients. More precise methods for assessing pain in MS are needed, and the roles of patient questionnaires, physical examination, bedside sensory testing, and quantitative sensory testing should be better defined. Moreover, more research is necessary to study the relationships between pain and depression, fatigue, and cognitive deficits. Certain types of pain associated with MS, such as pain related to optic neuritis and muscle spasms, have not been well characterized. Furthermore, there is limited understanding of how clinical exacerbations of MS relate to the occurrence, type, and intensity of pain. Identifying and characterizing the mechanisms behind different types of pain in MS patients, especially extremity pain, which is the most common type, is crucial for improved pain classification and the development of mechanism-based treatments. As the treatment of MS-related pain remains poorly understood, it is essential to conduct randomized controlled trials to evaluate the effectiveness and safety of existing treatments for neuropathic and non-neuropathic pain in MS patients. These treatments include antidepressants, anticonvulsants, opioid analgesics, non-steroidal anti-inflammatory drugs, and non-pharmacologic therapies. Additionally, few studies have explored the effects of disease-modifying therapy on pain in MS patients, not only in relieving pain but also as a potential source of pain. Given the widespread use of such therapy for relapsing MS, research on its effects on pain and the possibility of preventing pain should be prioritized in future clinical trials. Understanding the implications of pain treatment for patients, clinicians, policy-makers, and third-party payers is essential in determining the risk–benefit ratio and cost-effectiveness of various treatments [20,21,22,23].

Given the aging global population and the increasing prevalence of neurodegenerative disorders, untreated pain is becoming a significant burden for a growing number of individuals. It is crucial to establish a global consensus on strategies to address these challenges, enabling large-scale, high-quality clinical trials in the future. This will pave the way for the development of pain interventions based on a thorough understanding of the underlying mechanisms, ultimately leading to an improved quality of life for many people living with neurodegenerative disorders [20,21,22,23].

#### 4.2.4. COPD

It is crucial to establish standardized assessment tools for measuring pain in COPD patients. Future studies should focus on determining the precise prevalence of pain in relation to disease severity and comorbidity. Additionally, research should delve into the causes, progression, and characteristics of pain, and clinical intervention trials should be conducted. Moreover, it is vital to improve pain recognition and treatment in clinical practice. Integrating pain assessment into regular comprehensive symptom evaluations for COPD patients is important. It is also essential to discuss the prevalence of pain and its potential impact on the quality of life in COPD guidelines to raise awareness and enhance recognition of this aspect of the disease [29].

#### 4.2.5. CKD

It is necessary to extend a routine symptom assessment to patients with earlier stages of CKD as well. Renal and POS-renal are simple assessment tools that screen for common symptoms experienced by CKD patients and offer the opportunity to shift care towards a more patient-centered approach. Furthermore, more comprehensive pain assessment tools validated for CKD patients are available, such as the Visual Analog Scale (VAS), Verbal Rating Scale (VRS), and Numeric Rating Scale (NRS).

## 5. Conclusions

The prevalence of pain is high in patients with cancer, COPD, multiple sclerosis, Huntington’s disease, Parkinson’s disease, Alzheimer’s disease, and traumatic brain injury, as well as heart, liver, and kidney failure. The majority of patients who suffer from any of these chronic diseases are especially at high risk of developing pain at later stages. Pain does not receive enough attention and is not properly managed. In some cases, pain was reported even more frequently than the principal presenting symptoms of these diseases. Future studies are warranted to establish the prevalence of chronic pain more precisely and develop better methods of pain screening, detection, and management. More studies are also required to better understand the pathophysiology and mechanisms of chronic pain in some diseases, especially in neurodegenerative diseases. It would be also important to provide clear definitions of pain, such as acute, subacute, or chronic and its duration. Existing pain management methods should be widely applied in clinical practice, especially if solid evidence of their efficacy is established (e.g., regional nerve blocks to prevent and manage postamputation pain; opioids, regional anesthesia, pumps for cancer-related pain). For some patient populations, such as nursing home residents, in patients with dementia and neurodegenerative diseases, the most limiting factor for effective pain management is pain detection. Therefore, apart from better use of existing standard pain screening, detection, and rating, developing newer pain biomarkers could probably improve the quality of care. 

## Figures and Tables

**Table 1:** Characteristics of the included studies.

Author, Year, Citation	Location	Study Design	Diagnosis	Characteristics of Population	Number of Patients
Snijders 2023 [16]	Head and neck: Europe, Asia, North America Bronchus/lung: Europe, Asia, North America, Oceania Breast: all continents GI: Europe, Asia, North America, Oceania Prostate: Europe, Asia, North America, Oceania Gyn: Europe, Asia, North America, Oceania Hematological: Europe, Asia, North America, Oceania	SR + MA	Cancer: head and neck, bronchus/lung, breast, GI, prostate, Gyn, hematological	Cancer	Head and Neck: 4718 Bronchus/lung: 15,725 Breast: 52,376 GI: 19,911 Prostate: 4445 Gyn: 5924 Hematological: 4869
Haenen 2022 [17]	Breast: Asia, Europe Gyn: Europe, Oceania Lung: Europe, North America Prostate: Europe Rectal: South America	SR + MA	Cancer: breast, lung, Gyn, prostate, rectal	Solid cancer	Breast: 11,996 Lung: 588 Gyn: 337 Prostate: 109 Rectal: 40
Cole 2022 [18]	Finland, Netherlands, Italy, Norway, US, Hong Kong, Czech Republic, England, France, Germany, Israel, Japan, Brazil, Turkey, Austria, Sweden, Australia, China, Korea	SR	Arthritis, depression, dementia, osteoporosis, pressure ulcer, falls, anxiety, contracture	Nursing home residents	106–1,387,405
Evans 2022 [19]	Denmark, UK, Turkey, Scotland, US, Germany, Italy, Greece, India, Egypt, Finland	SR + MA	Amputation: cancer, trauma, vasculopathy	Residual limb pain	1347
Hurwitz 2021 [20]	Japan, US, Italy, Germany, Canada, France, Australia, Sweden, Finland, UK,	SR + MA	ALS	ALS	1426
Innes 2020 [21]	OA: Taiwan, US Fibromyalgia: Taiwan Headache: Taiwan, Norway, Canada	SR	Dementia	OA, fibromyalgia, headache, migraine, Alzheimer’s	701,593 OA: 201,495 Fibromyalgia: 165,000 Headache: 319,023
Chang 2021 [22]	NG	SR	Neurodegenerative diseases with atypical parkinsonism	MSA, PSP, CBS, DLB, FTD	NG
Sprenger 2019 [23]	UK, Germany, France, US, Italy, Cyprus, Norway	SR + MA	HD	HD	2578
Rana 2019 [24]	Sweden, UK, Austria, Italy, Spain, Israel, Germany, US, Canada, China	SR + MA	Atypical parkinsonism	MSA, PSP, CBS, LBD	CBS: 55 LBD: 95 MSA: 599 PSP: 242
Delwel 2017 [25]	UK, Brazil, Australia, US, Denmark, Switzerland, Malaysia, Norway, Japan, Turkey, Greece, Thailand, Finland, Germany	SR	Dementia	Dementia	257 (focused on pain)
Drageset 2014 [26]	US (no information on all the studies)	SR	Cancer: colon	Nursing Home Residents with Cancer	277,249
Stubbs 2014 [27]	US, UK, Australia, Italy, Japan, China, Nigeria, Netherlands, Taiwan, Turkey	SR + MA	Chronic pain	Seniors	32,705 for narrative, 17,926 for MA
Alemzadeh-Ansari 2017 [28]	NG	SR	CHF	CHF	20,875
van Isselt 2014 [29]	US, UK, Canada, Norway, Netherlands	SR + MA	COPD	COPD	12,479
Lee 2015 [30]	Norway, Canada, US	SR	COPD	COPD	8677
Van den Beuken-van Everdingen 2007 [31]	All continents	SR	Cancer: head and neck, GI, lung/bronchus, breast, urogenital, Gyn	Cancer	Head and neck: 95 GI: 564 Lung/bronchus: 1546 Breast: 420 Urogenital: 336 Gyn: 372
Davison 2021 [32]	US, Saudi Arabia, Norway, Republic of Guinea, Italy, Morocco, Australia, Spain, Republic of Korea, Hong Kong, Canada, Brazil, Turkey, Israel, Uruguay, Switzerland, Lebanon, Taiwan, Poland, UK, India, Netherlands, Iran, Sri Lanka, Malaysia, Germany, China	SR + MA	CKD	CKD	16,558
Lambourg 2021 [33]	US, UK—most common	SR + MA	CKD	CKD	40,678
Brkovic 2016 [34]	Scotland, Italy, Kuwait, Brazil, US, Canada, Morocco, Spain, UK, Serbia, Turkey, Switzerland, Israel, Pakistan, Japan, Iran, France	SR	ESRD	ESRD patients on hemodialysis	6917
Murtagh 2007 [35]	NG	SR	ESRD	ESRD	462 for pain only
Peng 2019 [36]	North America, Europe, Asia, Africa	SR + MA	End-stage liver disease	End-stage liver disease	5434
Nampiaparampil 2008 [37]	NG	SR	TBI	TBI	Headache: 1670 Chronic pain among civilians: 3289 Chronic pain among veterans: 917
Harriott 2020 [38]	Europe, Asia, North America	SR + MA	Ischemic stroke	Ischemic stroke	33,231
Liampas 2020 [39]	NG	SR + MA	Stroke	Stroke	20,668
O’Connor 2008 [40]	Belgium, Denmark, Canada, Italy, Norway, Sweden	SR	MS	MS	3360

Abbreviations: ALS—amyotrophic lateral sclerosis; CBS—cortico-basal syndrome; CHF—chronic heart failure; CKD—chronic kidney disease; COPD—chronic obstructive pulmonary disease; CPSP—chronic postsurgical pain; CVD—cardiovascular disease; DLB—dementia with Lewy bodies; ESRD—end-stage renal disease; FTD—frontotemporal dementia; GI—gastrointestinal; Gyn—gynecological; HD—Huntington disease; HF—heart failure; LBD—Lewy body dementia; MA—meta-analysis; MS—multiple sclerosis; MSA—multiple system atrophy; MSK—musculoskeletal; NG—not given; OA—osteoarthritis; PSP—progressive supranuclear palsy; QoL—quality of life; SR—systematic review; TBI—traumatic brain injury.

**Table 2:** Prevalence and mechanisms of pain.

Author, Year, Citation	Reported Prevalence	Prevalence of Other Symptoms	Characteristics of Pain: (a) Organ, (b) Type	Reported Mechanism of Pain	Conclusions
Snijders 2023 [16]	Head and neck: 8.8–77.3% Bronchus/lung: 21–77% Breast: 4.2–91.2% GI: 6.1–87% Prostate: 12.6–39.1% Gyn: 6–69% Hematological: 8–86.9%	NG	(a) Head and neck, bronchus/lung, breast, GI, prostate, Gyn	NG	The prevalence of pain is high, especially with advanced stage
Haenen 2022 [17]	Breast: 49% (95% CI 40–58%) Lung: 39% (95% CI 11–73%) Gyn: 55% (95% CI 49–62%)	NG	(a) Breast, lung, Gyn, prostate, rectal (b) Neuropathic	Not reported in studies	At least half of cancer patients experience pain at least three months after therapy
Cole 2022 [18]	Current pain: 22.2–85% Persistent pain: 19.5–58.5% Chronic pain: 55.9–58.1%	NG	-	Depression can lower the pain threshold, or chronic pain can cause mood disorders	Many factors can affect pain, especially depression
Evans 2022 [19]	1 w: 50% 1 mo: 11% 3 mo: 23% 6 mo: 27% 1 y: 22% 2 y: 24%	NG	(a) Upper and lower limbs (b) Neuropathic pain	Nerve injuries	Upper limb amputations due to cancer and trauma are the most painful
Hurwitz 2021 [20]	60% (95% CI = 50–69%)	NG	(a) Head, neck, trunk, back—24.8% Upper limbs—41.5% Lower limbs—33.7% (b) Neuropathic	Multifactorial pain, causes include inadequate posture	Pain in ALS is high
Innes 2020 [21]	Not the focus	Prevalence of dementia OA: 7.5% Fibromyalgia: 3.8% Headache: 1.5%	(a) Head	Lifestyle, a vicious cycle between pain and cognitive decline	Dementia might lead to the development of chronic pain
Chang 2021 [22]	MSA: 40–81% PSP: 25–68% CBS: 14–83% DLB: 25–70% FTD: 33–58%	NG	(a) MSA: neck, back, limbs PSP: limbs, neck, back CBS: limbs DLB: multiple places FTD: head, neck, shoulder, abdomen (b) MSA: MSK, neuropathic, dystonic PSP: MSK CBS: dystonic DLB: neuropathic, MSK FTD: MSK	NG	Neurodegenerative diseases can lead to chronic pain. However, prevalence might be underestimated due to the cognitive decline of patients
Sprenger 2019 [23]	41.3% (95% CI: 36–46%) Range: 10–75%	SF-36 score is 84 (95% CI: 81–86%)	NG	Many confounding factors	Pain might be one of the HD symptoms. Pain burden in the HD population is lower than that in the general population
Rana 2019 [24]	CBS: 25% LBD: 38% MSA: 73% PSP: 52%	NG	(a) LBD: multilocalized (b) MSA: MSK, neuropathic, central, radicular, arthritis PSP: neuropathic, MSK, central, arthritis CBS: dystonic, central, MSK LBD: MSK	Damage of pain receptors, improper posture	Patients with atypical parkinsonism experience pain, especially in the limbs
Delwel 2017 [25]	7.4–21.7%	NG	(a) Orofacial	NG	Patients with dementia have poorer oral health. No conclusions on orofacial pain can be drawn
Drageset 2014 [26]	37–60% (not all the studies reported pain)	NG	NG	NG	High prevalence of pain in cancer patients
Stubbs 2014 [27]	32.5% (not the focus)	Prevalence of falls: 50.5%	(a) Lower limb pain	Not the focus	Seniors with pain are likely to fall, especially with foot pain
Alemzadeh-Ansari 2017 [28]	23–85%	NG	(a) Chest (b) Neuropathic	Ischemia, inflammation, ascites, constipation	Pain is common in CHF and is poorly controlled
van Isselt 2014 [29]	32–60%	FEV_1_% 21–48%	(a) Shoulders and neck, lumbar region, chest	NG	Patients with moderate COPD have increased pain
Lee 2015 [30]	66% (95% CI 44–85%)	NG	(a) Shoulder, neck, upper limb, chest, diffuse, lower limb, head, buttocks, back	Altered respiration mechanics	COPD patients with pain have worse symptoms and QoL
Van den Beuken-van Everdingen 2007 [31]	Head and neck: 70 (51–88%) GI: 59 (44–74%) Lung/bronchus: 55 (44–67%) Breast: 54 (44–64%) Urogenital: 52 (40–60%) Gyn: 60 (50–71%)	NG	(a) Head and neck, GI, lung/bronchus, breast, urogenital, Gyn	NG	Cancer pain is common
Davison 2021 [32]	Moderate or severe pain: 43.6% Chronic pain in hemodialysis patients: 60.5%	NG	(a) Bone, joint, muscle, widespread, lower extremities, neck and shoulders, back, upper extremities (b) MSK, neuropathic, atheropathic	NG	Chronic pain is common in CKD
Lambourg 2021 [33]	Overall: 60 (95% CI 56–64%) Chronic pain: 48 (95% CI 42–55%) Neuropathic pain: 10 (95% CI 6–15%) Transplant recipients: 46 (95% CI 37–56%) Dialysis: 63% (95% CI 55–70%) Non-dialysis: 63 (95% CI 55–70%)	NG	(a) Abdominal, headache, joint, bone (b) Neuropathic, MSK	Postsurgical, drug toxicity, mineral turnover	CKD patients experience severe pain
Brkovic 2016 [34]	Acute: 82% Chronic: 92%	NG	(a) Most—headache, MSK, diffuse (b) Ischemic, neuropathic	Probably comorbidities, procedural pain	High prevalence of pain in CKD patients
Murtagh 2007 [35]	47% (8–82%)	Fatigue 71% (12–97%), pruritus 55% (10–77%), constipation 53% (8–57%), anorexia 49% (25–61%), sleep disturbance 44% (20–83%), anxiety 38% (12–52%), dyspnea 35% (11–55%), nausea 33% (15–48%), restless legs 30% (8–52%), depression 27% (5–58%)	(a) Muscle cramps, headache	NG	(Not related to pain)
Peng 2019 [36]	30–79%	Breathlessness 20–88%, muscle cramps 56–68%, insomnia 26–77%, daytime sleepiness 29.5–71%, depression 4.5–64%, anxiety 14–45%, erectile dysfunction 53–93%	NG	NG	Symptoms of end-stage liver disease are similar to the symptoms of other advanced diseases
Nampiaparampil 2008 [37]	Headache: 57.8 (95% CI 55.5–60.2%) Chronic pain among civilians: 51.5 (95% CI 49.8–53.2%) Chronic pain among veterans: 43.1 (95% CI 39.9–46.3%)	NG	(a) Headache	Brain injury	Chronic pain is common in TBI
Harriott 2020 [38]	6–44%	NG	(a) Headache	Changes in innervation, increases in compartmental pressures, meningeal inflammation	Headache is common after an ischemic stroke
Liampas 2020 [39]	Overall: 11 (95% CI 7–18%) Stroke onset: 26% (95% CI 18–35%); 1mo: 31% (95% CI 22–42%); 1 mo-1y: 41% (95% CI 33.9–49.0%); 1y+: 5% (95% CI 3–8%)	NG	(b) Neuropathic	NG	Neuropathic pain is common up to one year poststroke
O’Connor 2008 [40]	29–86%	NG	(a) Extremity, back pain, headache, trigeminal neuralgia (b) Neuropathic, MSK, mixed	Psychosocial factors	Extremity pain is the most common in MS

Abbreviations: ALS—amyotrophic lateral sclerosis; CBS—cortico-basal syndrome; CHF—chronic heart failure; CI—confidence interval; CKD—chronic kidney disease; COPD—chronic obstructive lung disease; DLB—dementia with Lewy bodies; FEV_1_—forced expiratory volume; FTD—frontotemporal dementia; GI—gastrointestinal; Gyn—gynecological; HD—Huntington’s disease; LBD—Lewy body dementia; mo—month; MS—multiple sclerosis; MSA—multiple system atrophy; MSK—musculoskeletal; NG—not given; OA—osteoarthritis; SF-36—36-Item Short Form Survey; TBI—traumatic brain injury; w—week; y—year.

**Table 3:** AMSTAR quality assessment.

Author, Citation	1	2	3	4	5	6	7	8	9	10	11	12	13	14	15	16
Snijders 2023 [16]	+	-	-	PY	+	-	-	PY	PY	-	+	-	-	-	-	+
Haenen 2022 [17]	+	PY	-	PY	+	-	-	PY	PY	-	+	-	-	+	-	+
Cole 2022 [18]	+	-	-	PY	+	-	-	PY	PY	-	N/A	N/A	-	N/A	-	+
Evans 2022 [19]	+	+	-	PY	+	+	-	PY	PY	-	+	-	-	-	-	+
Hurwitz 2021 [20]	+	-	-	PY	-	-	-	PY	PY	-	+	-	-	+	-	+
Innes 2020 [21]	+	-	+	PY	-	-	-	+	PY	-	+	-	-	-	-	+
Chang 2021 [22]	+	-	-	PY	-	-	-	-	-	-	N/A	N/A	-	N/A	-	+
Sprenger 2019 [23]	+	+	-	PY	+	-	-	PY	PY	-	+	-	-	-	-	-
Rana 2019 [24]	+	-	-	PY	+	-	-	PY	-	-	+	-	-	+	+	+
Delwel 2017 [25]	+	-	-	PY	+	-	+	PY	PY	-	N/A	N/A	-	N/A	-	+
Drageset 2014 [26]	+	-	-	PY	+	-	-	PY	PY	-	N/A	N/A	-	N/A	-	+
Stubbs 2014 [27]	+	-	-	PY	+	-	-	+	PY	-	+	-	-	+	+	+
Alemzadeh-Ansari 2017 [28]	+	-	-	PY	-	-	-	PY	-	-	N/A	N/A	-	N/A	-	-
van Isselt 2014 [29]	+	-	-	PY	+	+	-	PY	PY	-	+	-	-	-	-	+
Lee 2015 [30]	+	+	-	PY	+	-	-	PY	PY	-	N/A	N/A	-	N/A	-	+
Van den Beuken [31]	+	-	+	PY	-	-	-	PY	PY	-	+	-	-	+	-	-
Davison 2021 [32]	+	+	-	-	+	+	+	PY	PY	-	+	-	-	+	+	+
Lambourg 2021 [33]	+	+	-	PY	+	-	-	PY	PY	-	+	-	-	+	+	+
Brkovic 2016 [34]	+	+	-	PY	+	-	-	PY	PY	-	N/A	N/A	-	N/A	-	+
Murtagh 2007 [35]	+	-	+	+	-	-	-	PY	-	-	N/A	N/A	-	N/A	-	+
Peng 2019 [36]	+	-	-	PY	+	-	-	-	PY	-	+	-	-	-	-	+
Nampiaparampil 2008 [37]	+	-	+	+	-	-	-	PY	-	-	+	-	-	-	-	+
Harriott 2020 [38]	+	-	-	PY	+	-	-	PY	-	-	+	-	-	+	+	+
Liampas 2020 [39]	+	+	+	PY	+	+	-	-	PY	-	+	-	+	-	+	+
O’Connor 2008 [40]	+	-	-	+	-	-	-	+	-	-	N/A	N/A	-	N/A	-	+
Did the research questions and inclusion criteria for the review include the components of PICO? Did the report of the review contain an explicit statement that the review methods were established prior to the conduct of the review and did the report justify any significant deviations from the protocol? Did the review authors explain their selection of the study designs for inclusion in the review? Did the review authors use a comprehensive literature search strategy? Did the review authors perform study selection in duplicate? Did the review authors perform data extraction in duplicate? Did the review authors provide a list of excluded studies and justify the exclusions? Did the review authors describe the included studies in adequate detail? Did the review authors use a satisfactory technique for assessing the risk of bias (RoB) in individual studies that were included in the review? Did the review authors report on the sources of funding for the studies included in the review? If a meta-analysis was performed, did the review authors use appropriate methods for statistical combination of results? If meta-analysis was performed, did the review authors assess the potential impact of RoB in individual studies on the results of the meta-analysis or other evidence synthesis? Did the review authors account for RoB in individual studies when interpreting/discussing the results of the review? Did the review authors provide a satisfactory explanation for, and discussion of, any heterogeneity observed in the results of the review? If they performed quantitative synthesis did the review authors carry out adequate investigation of publication bias (small study bias) and discuss its likely impact on the results of the review? Did the review authors report any potential sources of conflict of interest, including any funding they received for conducting the review?																

Abbreviations: MA—meta-analysis; N/A—not applicable; PY—partial yes; SR—systematic review; RoB—risk of bias.

## Data Availability

No new data were created or analyzed in this study. Data sharing is not applicable to this article.

## Supplementary Materials

The following supporting information can be downloaded at: https://www.mdpi.com/article/10.3390/jcm12237302/s1, File S1: Search terms.
