# Supplementary material for: The Prevalence of Pain in Chronic Diseases: An Umbrella Review of Systematic Reviews
Source: J Clin Med. 2023 Nov 25;12(23):7302. doi: 10.3390/jcm12237302 (PMC10707436; doi:10.3390/jcm12237302)
Supplement: Supplementary file 1 [file jcm-12-07302-s001.zip › jcm-2680853-supplementary.pdf]

Search terms (Prevalence of chronic pain in different diseases.

(("pain"[MeSH Terms] OR "pain"[All Fields]) AND ("epidemiology"[MeSH Subheading] OR "epidemiology"[All Fields] OR "prevalence"[All Fields] OR "prevalence"[MeSH Terms] OR "prevalance"[All Fields] OR "prevalences"[All Fields] OR "prevalence s"[All Fields] OR "prevalent"[All Fields] OR "prevalently"[All Fields] OR "prevalents"[All Fields])) AND (clinicaltrial[Filter])

Pain prevalence in cancer

(("pain"[MeSH Terms] OR "pain"[All Fields]) AND ("epidemiology"[MeSH Subheading] OR "epidemiology"[All Fields] OR "prevalence"[All Fields] OR "prevalence"[MeSH Terms] OR "prevalance"[All Fields] OR "prevalences"[All Fields] OR "prevalence s"[All Fields] OR "prevalent"[All Fields] OR "prevalently"[All Fields] OR "prevalents"[All Fields]) AND ("cancer s"[All Fields] OR "cancerated"[All Fields] OR "canceration"[All Fields] OR "cancerization"[All Fields] OR "cancerized"[All Fields] OR "cancerous"[All Fields] OR "neoplasms"[MeSH Terms] OR "neoplasms"[All Fields] OR "cancer"[All Fields] OR "cancers"[All Fields])) AND (clinicaltrial[Filter])

647

Pain prevalence in neurodegenerative diseases

(pain prevalence) AND (neurodegenerative diseases) Filters: Clinical Trial Sort by: Most Recent (("pain"[MeSH Terms] OR "pain"[All Fields]) AND ("epidemiology"[MeSH Subheading] OR "epidemiology"[All Fields] OR "prevalence"[All Fields] OR "prevalence"[MeSH Terms] OR "prevalance"[All Fields] OR "prevalences"[All Fields] OR "prevalence s"[All Fields] OR "prevalent"[All Fields] OR "prevalently"[All Fields] OR "prevalents"[All Fields]) AND ("neurodegenerative diseases"[MeSH Terms] OR ("neurodegenerative"[All Fields] AND "diseases"[All Fields]) OR "neurodegenerative diseases"[All Fields])) AND (clinicaltrial[Filter])

37

Pain prevalence in multiple sclerosis

(pain prevalence) AND (multiple sclerosis) Filters: Clinical Trial Sort by: Most Recent (("pain"[MeSH Terms] OR "pain"[All Fields]) AND ("epidemiology"[MeSH Subheading] OR "epidemiology"[All Fields] OR "prevalence"[All Fields] OR "prevalence"[MeSH Terms] OR "prevalance"[All Fields] OR "prevalences"[All Fields] OR "prevalence s"[All Fields] OR "prevalent"[All Fields] OR "prevalently"[All Fields] OR "prevalents"[All Fields]) AND ("multiple sclerosis"[MeSH Terms] OR ("multiple"[All Fields] AND "sclerosis"[All Fields]) OR "multiple sclerosis"[All Fields])) AND (clinicaltrial[Filter])

4

Pain prevalence in Parkinson disease

(pain prevalence) AND (parkinson) Filters: Clinical Trial Sort by: Most Recent (("pain"[MeSH Terms] OR "pain"[All Fields]) AND ("epidemiology"[MeSH Subheading] OR "epidemiology"[All Fields] OR

"prevalence"[All Fields] OR "prevalence"[MeSH Terms] OR "prevalance"[All Fields] OR "prevalences"[All Fields] OR "prevalence s"[All Fields] OR "prevalent"[All Fields] OR "prevalently"[All Fields] OR "prevalents"[All Fields]) AND ("parkinson disease"[MeSH Terms] OR ("parkinson"[All Fields] AND "disease"[All Fields]) OR "parkinson disease"[All Fields] OR "parkinsons"[All Fields] OR "parkinson"[All Fields] OR "parkinson s"[All Fields] OR "parkinsonian disorders"[MeSH Terms] OR ("parkinsonian"[All Fields] AND "disorders"[All Fields]) OR "parkinsonian disorders"[All Fields] OR "parkinsonism"[All Fields] OR "parkinsonisms"[All Fields] OR "parkinsons s"[All Fields])) AND (clinicaltrial[Filter])

25

Pain prevalence in patients with cerebrovascular diseases:

(pain prevalence) AND (cerebrovascular disease) Filters: Clinical Trial Sort by: Most Recent (("pain"[MeSH Terms] OR "pain"[All Fields]) AND ("epidemiology"[MeSH Subheading] OR "epidemiology"[All Fields] OR "prevalence"[All Fields] OR "prevalence"[MeSH Terms] OR "prevalance"[All Fields] OR "prevalences"[All Fields] OR "prevalence s"[All Fields] OR "prevalent"[All Fields] OR "prevalently"[All Fields] OR "prevalents"[All Fields]) AND ("cerebrovascular disorders"[MeSH Terms] OR ("cerebrovascular"[All Fields] AND "disorders"[All Fields]) OR "cerebrovascular disorders"[All Fields] OR ("cerebrovascular"[All Fields] AND "disease"[All Fields]) OR "cerebrovascular disease"[All Fields])) AND (clinicaltrial[Filter])

86

Pain prevalence in chronic heart failure:

(pain prevalence) AND (Chronic heart failure) Sort by: Most Recent ("pain"[MeSH Terms] OR "pain"[All Fields]) AND ("epidemiology"[MeSH Subheading] OR "epidemiology"[All Fields] OR "prevalence"[All Fields] OR "prevalence"[MeSH Terms] OR "prevalance"[All Fields] OR "prevalences"[All Fields] OR "prevalence s"[All Fields] OR "prevalent"[All Fields] OR "prevalently"[All Fields] OR "prevalents"[All Fields]) AND (("chronic"[All Fields] OR "chronical"[All Fields] OR "chronically"[All Fields] OR "chronicities"[All Fields] OR "chronicity"[All Fields] OR "chronicization"[All Fields] OR "chronics"[All Fields]) AND ("heart failure"[MeSH Terms] OR ("heart"[All Fields] AND "failure"[All Fields]) OR "heart failure"[All Fields]))

23

Pain prevalence in chronic kidney failure:

(pain prevalence) AND (chronic kidney failure) Sort by: Most Recent ("pain"[MeSH Terms] OR "pain"[All Fields]) AND ("epidemiology"[MeSH Subheading] OR "epidemiology"[All Fields] OR "prevalence"[All Fields] OR "prevalence"[MeSH Terms] OR "prevalance"[All Fields] OR "prevalences"[All Fields] OR "prevalence s"[All Fields] OR "prevalent"[All Fields] OR "prevalently"[All Fields] OR "prevalents"[All Fields]) AND ("kidney failure, chronic"[MeSH Terms] OR ("kidney"[All Fields] AND "failure"[All Fields] AND "chronic"[All Fields]) OR "chronic kidney failure"[All Fields] OR ("chronic"[All Fields] AND "kidney"[All Fields] AND "failure"[All Fields]))

19

Pain prevalence in chronic liver diseases

(chronic pain) AND (chronic liver disease) Filters: Systematic Review Sort by: Most Recent (("chronic pain"[MeSH Terms] OR ("chronic"[All Fields] AND "pain"[All Fields]) OR "chronic pain"[All Fields]) AND (("chronic"[All Fields] OR "chronical"[All Fields] OR "chronically"[All Fields] OR "chronicities"[All Fields] OR "chronicity"[All Fields] OR "chronicization"[All Fields] OR "chronics"[All Fields]) AND ("liver diseases"[MeSH Terms] OR ("liver"[All Fields] AND "diseases"[All Fields]) OR "liver diseases"[All Fields] OR ("liver"[All Fields] AND "disease"[All Fields]) OR "liver disease"[All Fields]))) AND (systematicreview[Filter])

53

Pain prevalence in nursing home residents

chronic pain AND nursing home residents Sort by: Most Recent ("chronic pain"[MeSH Terms] OR ("chronic"[All Fields] AND "pain"[All Fields]) OR "chronic pain"[All Fields]) AND (("nursing homes"[MeSH Terms] OR ("nursing"[All Fields] AND "homes"[All Fields]) OR "nursing homes"[All Fields] OR ("nursing"[All Fields] AND "home"[All Fields]) OR "nursing home"[All Fields]) AND ("internship and residency"[MeSH Terms] OR ("internship"[All Fields] AND "residency"[All Fields]) OR "internship and residency"[All Fields] OR "residencies"[All Fields] OR "residency"[All Fields] OR "reside"[All Fields] OR "resided"[All Fields] OR "residence"[All Fields] OR "residence s"[All Fields] OR "residences"[All Fields] OR "residency s"[All Fields] OR "resident"[All Fields] OR "resident s"[All Fields] OR "residents"[All Fields] OR "resides"[All Fields] OR "residing"[All Fields]))

230

Pain prevalence in patients after limb amputation:

(pain prevalence) AND (amputation) Sort by: Most Recent ("pain"[MeSH Terms] OR "pain"[All Fields]) AND ("epidemiology"[MeSH Subheading] OR "epidemiology"[All Fields] OR "prevalence"[All Fields] OR "prevalence"[MeSH Terms] OR "prevalance"[All Fields] OR "prevalences"[All Fields] OR "prevalence s"[All Fields] OR "prevalent"[All Fields] OR "prevalently"[All Fields] OR "prevalents"[All Fields]) AND ("amputate"[All Fields] OR "amputated"[All Fields] OR "amputating"[All Fields] OR "amputation, surgical"[MeSH Terms] OR ("amputation"[All Fields] AND "surgical"[All Fields]) OR "surgical amputation"[All Fields] OR "amputation"[All Fields] OR "amputations"[All Fields] OR "amputed"[All Fields])

45

Search terms (Prevalence of chronic pain in different diseases.
Pain prevalence in cancer: 647
Pain prevalence in neurodegenerative diseases: 37
Pain prevalence in multiple sclerosis: 4
Pain prevalence in Parkinson disease: 25
Pain prevalence in patients with cerebrovascular diseases: 86
Pain prevalence in chronic heart failure: 23
Pain prevalence in chronic kidney failure: 19
Pain prevalence in chronic liver diseases: 53
Pain prevalence in nursing home residents: 230
Pain prevalence in patients after limb amputation: 45
